# Longitudinal Neuropsychological Assessment in Two Elderly Adults With Attention-Deficit/Hyperactivity Disorder: Case Report

**DOI:** 10.3389/fpsyg.2019.01119

**Published:** 2019-05-28

**Authors:** Margarete Klein, Maria Aparecida Silva, Gabriel Okawa Belizario, Cristiana Castanho de Almeida Rocca, Antonio De Padua Serafim, Mario R. Louzã

**Affiliations:** ^1^Department of Psychiatry, Faculty of Medicine, University of São Paulo, São Paulo, Brazil; ^2^Institute of Psychiatry, Clinical Hospital, Faculty of Medicine, University of São Paulo, São Paulo, Brazil; ^3^Clinical Hospital, Faculty of Medicine, University of São Paulo, São Paulo, Brazil; ^4^Department of Psychology, Methodist University of São Paulo, São Bernardo do Campo, Brazil

**Keywords:** attention-deficit/hyperactivity disorder, elderly, neuropsychological assessment, cognitive decline, longitudinal evaluation

## Abstract

The neuropsychological deficits in attention-deficit/hyperactivity disorder (ADHD) may present clinical features similar to mild and/or major neurocognitive disorder and may act as a confounding factor, making it difficult to detect cognitive decline. In this paper, we present the results of longitudinal neuropsychological evaluations in two elderly women with ADHD. Three neuropsychological assessments were performed in two women with ADHD (60 and 77 years old) between 2010 and 2013 at intervals varying from 12 to 15 months. We used structural magnetic resonance imaging to rule out significant abnormalities that could account for cognitive impairment. The results showed two different cognitive profiles with fluctuations in performance over these 2 years, sometimes with improvement and sometimes with decline of some functions such as attention, memory, inhibitory control, and reaction time. To minimize confounding aspects of these fluctuations in clinical practice, we used a longer follow-up with the application of a reliable change index and a minimum of three spaced assessments to provide a more consistent baseline cognitive profile. Our findings did not indicate a consistent cognitive decline, suggesting a less pessimistic perspective about cognitive impairments that could be a prodrome of ADHD-related dementia.

## Introduction

According to the American Psychiatric Association (American Psychiatric Association [APA], 2013), attention-deficit/hyperactivity disorder (ADHD) is a complex neuropsychiatric disorder with a persistent pattern of inattention and/or hyperactivity–impulsivity throughout the lifetime. The symptoms interfere with development or functionality in multiple areas of life, such as in academia and relationships as well as at work. This disorder occurs in most cultures in approximately 5% of children and 2.5% of adults (American Psychiatric Association [APA], 2013); a prevalence of approximately 3% in older people has been described (Guldberg-Kjär and Johansson, 2009; Michielsen et al., 2012).

Although studies show an age-dependent decline in ADHD symptoms (Michielsen et al., 2012; Das et al., 2014), the persistence of symptoms might also lead to significant impairment in older age with a cumulative impact (Kooij et al., 2016; Srinivasan et al., 2016). Moreover, multiple domains of neurocognitive impairment such as executive function, attention, memory, and language are common in children (Sjöwall et al., 2013, 2015; Martino et al., 2017; Salari et al., 2017; Fabio et al., 2018; Shephard et al., 2018; Sjöwall and Thorell, 2019), adolescents (Fried et al., 2016; Steward et al., 2017; Weyandt et al., 2017), and young adults with ADHD (Faraone et al., 2015; Mostert et al., 2015; Antshel et al., 2016; Salomone et al., 2016). Recent studies suggest that some of these impairments may persist in older adults (Fuermaier et al., 2013; Thorell et al., 2017; Coelho et al., 2018).

There is large variability in cognitive expression among younger individuals and older adults with ADHD compared to healthy controls, ranging in individuals with ADHD from a complete lack of deficient scores to severe impairment (Mostert et al., 2015; Thorell et al., 2017). In addition, psychiatric disorders, such as depression and anxiety, are frequently comorbid with ADHD, which remains true for older adults (Michielsen et al., 2013; Das et al., 2014). The overlapping of symptoms can increase the patients’ cognitive difficulties and further complicate the diagnosis of this disorder (Michielsen et al., 2013).

In current psychogeriatric practice, ADHD still goes unrecognized. Existing cognitive deficits may present clinical features similar to mild and/or major neurocognitive disorder and may also act as a confounding factor, although these conditions differ from ADHD by their late onset (American Psychiatric Association [APA], 2013). However, clinicians need to pay extra attention to possible cognitive decline and dementia (Kooij et al., 2016). ADHD shares some similarities with the cognitive changes that come with aging, such as increased attentional vulnerability and less efficient memory (Harada et al., 2013), which may cause increased impairment in daily functioning in older individuals with ADHD (Thorell et al., 2017).

In relation to symptoms across the adult life span, studies have analyzed the association between ADHD and mild cognitive impairment (MCI, currently mNCD) and dementia. Golimstok et al. (2010) found a higher risk of dementia with Lewy bodies in patients with ADHD symptoms prior to adulthood, while a study conducted by Ivanchack et al. (2012) found no association between ADHD and mNCD or dementia. More recently, Fluegge and Fluegge (2018) found an association between antecedent ADHD (severe ADHD phenotype) and dementia subtype risk [Lewy body dementia (LBD) and Alzheimer’s disease (AD)]. They suggest that these relationships may be dependent upon the extent of metabolic dysregulation since controlling the analyses for diabetes, the significant association between antecedent ADHD and risk of AD does not remain, but it remains for LBD.

In the elderly population, neuropsychological measures have proven to be important tools to help clinicians identify cognitive and functional profiles that can differentiate the transition from benign cognitive aging to dementia (Fichman et al., 2013). However, due to the factors discussed above regarding ADHD, finding patterns of change that may indicate earlier cognitive decline in older ADHD is a great challenge. Considering the heterogeneity in cognitive expression in patients with ADHD, assessments are a useful tool to verify individual differences and measure changes in cognitive functioning over time, distinguishing changes that may be clinically relevant in patients with ADHD (Lange et al., 2014). Thus, because results from longitudinal studies are not yet available, case reports may initially be more useful for providing preliminary insights and discussion about patterns of cognitive functioning over the long term in this age group that is common in our clinical practice. To our knowledge, there are no prospective studies examining cognitive performance in older adults with ADHD, with or without treatment, evaluated with a comprehensive battery of cognitive tests.

The aim of the present case reports was to investigate the presence of cognitive decline and to identify other neuropsychological characteristics in elderly subjects diagnosed with ADHD.

## Case Report

Participants signed a free and informed consent form consenting to participation in the research and publication of the data collected in a case report. The final format of this manuscript along with the authorizations for publication was submitted and approved by the ethics committee of the Hospital das Clínicas of the University of São Paulo, protocol number 3.118.878.

### Case 1

Mrs. G.F., 60 years old, married, oceanographer, civil servant. She exhibited behaviors such as inattentiveness and forgetfulness dating back to childhood, when they were associated with poor performance at school to the point where she had to repeat grade 1. In adulthood, she exhibited significant functional impairment due to the inability to self-organize or prioritize tasks, a tendency to procrastinate, and a need for silence to concentrate and be productive. Eventually, she resigned from a management position at work because she was not able to finish assignments on time and had to take work home, which affected her family life. Psychiatric care was initially sought due to a depressive episode with persistence, even after remission, of the following symptoms: inattentiveness, forgetfulness, difficulty falling asleep, delays meeting her commitments, and lack of planning.

#### Medical History

No family history of dementia and no clinical problems were reported at the first assessment. Neuroimaging exams (MRI) revealed normal morphology and size for the patient’s age group. There was no evidence of acute ischemic injury.

#### Diagnosis

Attention-deficit/hyperactivity disorder (inattentive subtype) and depressive disorder (remitted). The proposed medical treatment was venlafaxine 75 mg/day and methylphenidate up to 60 mg/day.

### Case 2

Mrs. T.B, 77 years old, widowed, four completed years of formal schooling, retired. In 2001, she was 67 years old and healthy; however, a friend observed that she was quite absent-minded and advised her to seek help. The patient reported that she was agitated and forgetful during childhood and was the only one of three siblings who failed to complete a higher education. As a child, she was restless, used to escape from school to play, and did not pay attention when she was in the classroom. As a consequence, she often failed school assignments, needed to repeat some school years, and dropped out of school in her early teens. She worked many years for a company where the work was mechanical and repetitive, and she rarely arrived on time at work, missed appointments, and was less efficient than her colleagues. She never read an entire book because of her difficulty concentrating. She always forgot to pay bills, lost or misplaced personal objects, and needed the help of her family to remember commitments. She married at 20, and her husband took care of everything. After he died, her everyday life was seriously affected. Eventually, her children had to assume the task of organizing her life. Some years later, before treatment, she left home forgetting a roast in the oven.

#### Medical History

At the first assessment, in 2001, the patient reported no clinical problems, no signs of depression or anxiety, and denied having ever experienced any psychiatry conditions. At that assessment, an electrocardiogram (ECG; results were within the limits of normality) and a computerized tomography scan of the brain (presented as preserved, with normal attenuation values to X-rays) were collected.

#### Diagnosis

Attention-deficit/hyperactivity disorder (inattentive subtype). The proposed medical treatment was methylphenidate up to 10 mg/day. In the period leading up to the last evaluation, she took methylphenidate 20 mg/day.

### Procedures

Participants were treated at the hospital by psychiatrists and neuropsychologists of the Attention Deficit Hyperactivity Disorder Program (PRODATH) and followed up for 2 years. The assessment included a complete clinical history and clinical examination. ADHD diagnosis was performed according to the *Diagnostic and Statistical Manual of Mental Disorders*, fourth edition (American Psychiatric Association [APA], 2013), criteria, and comorbidities were investigated. Both participants were subjected to structural magnetic resonance imaging (MRI) to rule out significant abnormalities that could account for cognitive impairment and/or act as confounding factors. Laboratory tests and an ECG were also performed. Estimated intellectual quotient (IQ) (Ringe et al., 2002) and Mini-Mental State Examination (MMSE) (Folstein et al., 1983) measures were collected in 2010 during the first neuropsychological evaluation. Responses to the Informant Questionnaire on Cognitive Decline in the Elderly (IQCODE) (Jorm, 1994) were collected only at the third assessment. In addition, we used the Geriatric Depression Scale (GDS) (Yesavage et al., 1983) and Beck Anxiety Inventory (BAI) (Beck et al., 1988).

At the time of assessment, the participants were requested to interrupt their medication (methylphenidate) for 24 h, and the Case 1 participant continued use of an antidepressant medication. The participants signed an informed consent form allowing the use of their clinical and neuropsychological data.

#### Neuropsychological Evaluation

Three neuropsychological assessments were performed from 2010 to 2013 at intervals varying from 12 to 15 months. The ages of the participants refer to age at neuropsychological baseline assessed in 2010. Each assessment comprised two sessions lasting approximately 90 min each. The instruments used in the neuropsychological evaluation can be found in Table 1 (Supplementary Material). Scores were considered as impaired at or above 1.5 standard deviations (SDs) below typical performance (Albert et al., 2011) since there are no cutoff levels for *z*-scores suggested for ADHD (Mostert et al., 2015). To verify real significant change and/or decline over time, we used a statistical procedure called the reliable change index (RCI) (Jacobson and Truax, 1991). Values were considered significant if ≥1.96 (Supplementary Material).

**TABLE 1:** Cognitive tests administered and normative data.

**Cognitive tests**	**Domain assessed**
Vocabulary and Matrix Reasoning	Estimated intelligence Quotient (IQ)
Digit Span (forward and backward)	Attentional span Working memory
Rey Auditory Verbal Learning Test – RAVLT	Total immediate recall episodic memory (learning), late recall, and recognition of verbal materials
Brief Visual Memory Test – BVMT	Immediate recall episodic memory, late recall, and recognition of visual materials
Boston Naming Test	Naming skills (language)
Trail-Making Test – Parts A and B	Perceptual tracking of a sequence, speed performance, and divided attention, respectively
Stroop Test (scores from third card)	Inhibitory control
Wisconsin Card Sorting Test (WCST) – 64CV	Abstract reasoning ability Ability to shift cognitive strategies
Categorical and phonemic Verbal Fluency	Fluency (executive function) and speed of speech
Continuous Performance Test – CPT II	Sustained attention/alertness, impulsivity, and reaction time

*For detail on neuropsychological tests and the sources see Wechsler (1997), Spreen and Strauss, 1998, Lezak et al. (2004), and Strauss et al. (2006).*

#### Assessment of Rating Scales and Test Profile

##### Case 1

G.F. exhibited normal performance on the MMSE, with a score of 29 (within the cutoff point). The GDS and BAI, administered at all evaluations, did not indicate the presence of symptoms. The IQCODE indicated no changes/decline in cognition.

The clinical evaluation of the two cases can be found in the Supplementary Material.

**FIGURE 1:**
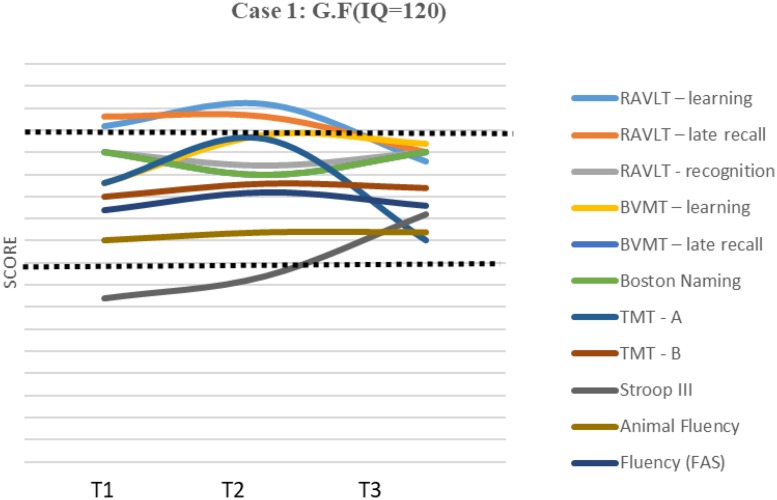
Results and evolution in *z*-scores of individual tests – Case 1. IQ, Intelectual quocient; T1, Time 1; T2, Time 2; T3, Time 3.

**FIGURE 2:**
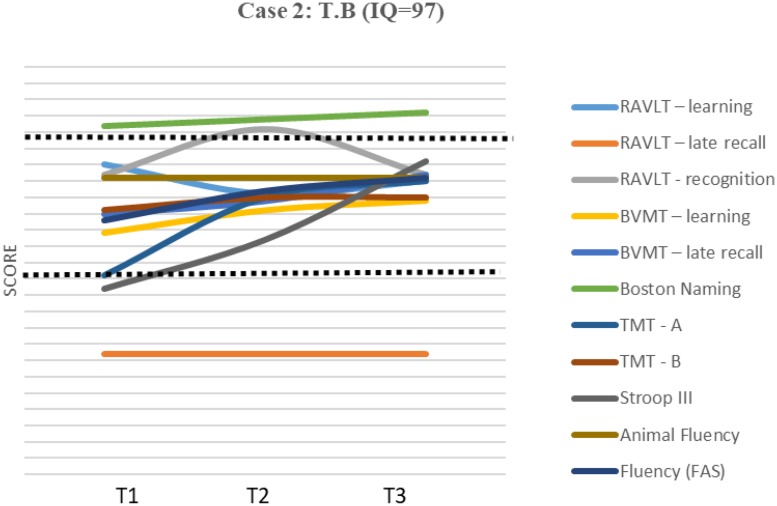
Results and evolution in *z*-scores of individual tests – Case 2. IQ, Intelectual quocient; T1, Time 1; T2, Time 2; T3, Time 3.

Test results are presented as *z*-scores (Table 2). A graphic representation of the scores and cognition evolution can be found in Figures 1, 2.

**TABLE 2:** Test’s results in *z* score and evaluation times.

**Tests**	**Case 1**			**Case 2**		
	**T1**	**T2**	**T3**	**T1**	**T2**	**T3**
RAVLT – total recall (learning)	1.6	2.1	0.8	0	−0.9	−0.5
RAVLT – late recall	1.8	1.8	1	−**5.8**	−**5.8**	−**5.8**
RAVLT – recognition	1	0.7	1	−0.3	1.1	−0.3
BVMT – total recall (learning)	0.3	1.4	1.2	−2.1	−1.4	−1.1
BVMT – late recall	−0.2	1.2	1.6	−**1.5**	−1.1	−0.3
Boston Naming	1	0.5	1	1.2	1.4	1.6
TMT – A	0.3	1.3	−1	−**3.4**	−1.0	−0.5
TMT – B	0	0.3	0.2	−1.4	−1	−1
Stroop (3)	−**2.3**	−**1.8**	−0.4	−**3.8**	−**2.3**	−0.1
Animal Fluency	−1	−0.8	−0.8	−0.4	−0.4	−0.4
Fluency (FAS)	−0.3	0.1	−0.2	−**1.7**	−0.8	−0.4

*Scores in bold are −1.5 *z* score; T1, Time 1; T2, Time 2; T3, Time 3; (3), Third.*

The results from the Continuous Performance Test II can be found in Table 3.

**TABLE 3:** Results from Continuous Performance Test – II.

**CPT-II**	**Case 1**			**Case 2**		
	**T1**	**T2**	**T3**	**T1**	**T2**	**T3**
CPT-Omissions (inattention)	A	GP	A	MA	MA	A
CPT-Commissions (impulsivity)	GP	A	A	A	A	GP
CPT – Reaction Time	468.05 LS	447.30 A	499.65 LS	506.45 AS	491.31 LS	490.91 LS
CPT – Vigilance	Poor	A	Poor	A	Poor	Poor

*T1, Time 1; T2, Time 2; T3, Time 3. A, Average; GP, good performance; MA, markedly atypical; AS, atypically slow; LS, a little slow.*

Case 1 showed normal performance in almost all tests (between -1 and +2.1 *z*-score), except in inhibitory control (-2.3 *z*-score).

This result improved from the first to the second evaluation and improved significantly from the second to the third evaluation, presenting within the expected range at the third evaluation. There were oscillations in performance from one evaluation to the next, but the RCI (Table 4) for specific cognitive domains, comparing T3 with T1, revealed an improvement in 6 of the 11 domains; the most consistent finding was a significant improvement in three of the domains {total learning, late recall/visual memory [Brief Visuospatial Memory Test (BVMT)], and inhibitory control (Stroop test—III)}. A decline in performance can be seen in total learning and late recall/verbal memory [Rey Auditory Verbal Learning Test (RAVLT) and Trial Making Test part A (TMT A)], but the differences are not significant. No changes were observed in RAVLT recognition and the Boston Naming Test.

**TABLE 4:** RCI between two times by case and test.

**Tests**	**Case 1**						**Case2**					
	**T2–T1**		**T3–T2**		**T3–T1**		**T2–T1**		**T3–T2**		**T3–T1**	
	**Min**	**Max**	**Min**	**Max**	**Min**	**Max**	**Min**	**Max**	**Min**	**Max**	**Min**	**Max**
RAVLT (total recall)	0.77	0.86	−1.42	−1.64	−1.03	−1.15	−1.23	−1.37	0.49	0.56	−0.61	−0.69
RAVLT (late recall)	0.00	0.00	−0.93	−1.08	−1.18	−1.32	0.00	0.00	0.00	0.00	0.00	0.00
RAVLT (recognition)	−0.46	−0.51	0.36	0.42	0.00	0.00	**2.02**	**2.26**	−1.60	−1.84	0.00	0.00
BVMT (total recall)	1.68	2.53	−0.21	−0.32	1.40	**2.11**	0.97	1.35	0.38	0.61	1.46	**2.02**
BVMT (late recall)	**2.11**	**3.17**	0.52	0.81	**2.81**	**4.23**	0.58	0.81	0.92	1.45	1.75	**2.42**
Boston Naming	−0.88	−1.35	1.15	1.15	0.00	0.00	0.39	0.60	0.51	0.51	0.77	1.19
TMT – A	1.04	1.19	**−2.43**	−**2.77**	−1.39	−1.58	**2.54**	**2.90**	0.46	0.53	**3.00**	**3.42**
TMT – B	0.35	0.38	−0.19	−0.21	0.16	0.17	0.42	0.45	0.00	0.00	0.49	0.53
Stroop III	1.15	1.15	**3.15**	**3.15**	**4.30**	**4.30**	**3.56**	**3.56**	**5.69**	**5.69**	**9.24**	**9.24**
Animal Fluency	0.33	0.33	0.00	0.00	0.33	0.33	0.00	0.00	0.00	0.00	0.00	0.00
Fluency (FAS)	0.60	0.60	−0.32	−0.32	0.24	0.24	1.11	1.11	0.56	0.56	1.67	1.67

*RCI in bold, statically significant at a 5% probability level.*

Alertness/sustained attention (CPT-II; Table 3) and the number of omission (inattention) and commission (impulsivity) errors were within the normal range in all three evaluations. Changes in the consistency of responses throughout the test [Hit RT (reaction time) Block Change (overall speed of correct answers throughout the test)] and/or lack of improvement in the reaction time over the course of the test [Hit SE (standard error) (measure of response speed consistency) Block Change] showed a predominantly “little slow” reaction time and poor vigilance.

##### Case 2

T.B. exhibited normal performance on the MMSE, with a score of 27 (above the cutoff point). The BAI and GDS, administered at all evaluations, did not indicate the presence of symptoms. The score on the IQCODE indicated a slight decline.

The patient’s neuropsychological profile (Table 2 and Figure 2) showed impaired performance in 6 of the 11 domains evaluated, with *z* < -3 in three of them [verbal late recall (RAVLT), attention involving perceptual tracking of a sequence and speed (TMT A), and inhibitory control (Stroop III)]. However, with the exception of her verbal late recall (RAVLT) score, which remained impaired (but stable), and verbal total recall (RAVLT learning), which declined (but not significantly), there was improved performance over time, with scores presenting within the normal range at the third evaluation. The RCI (Table 4) showed a significant improvement between T1 and T3 in four domains: visual total recall (learning) and late recall, Boston Naming Test, and inhibitory control (Stroop III). No change was observed in task animal fluency.

The alertness/sustained attention (CPT-II; Table 3) score was low, and changes in the consistency of responses were observed throughout the test, indicating that despite a small improvement in reaction time over the course of the test, the patient maintained poor vigilance.

## Discussion

Overall, the results of the case assessments showed impairments in cognitive functioning domains mainly related to attention, executive function (inhibitory control and phonemic fluency), and memory, and a predominance of slow reaction times in CPT. Although these cognitive findings do not characterize ADHD by themselves, they are consistent with previous literature (Hervey et al., 2004; Seidman, 2006; Kofler et al., 2013; Faraone et al., 2015; Mostert et al., 2015; Salomone et al., 2016; Weyandt et al., 2017), except for both participants’ performance in the working memory domain, which was average. This inconsistency with the literature may reflect possible variations (individual differences) that are found within a larger sample since ADHD in older adults was recently found to be associated with lower cognitive functioning in working memory (Semeijn et al., 2015; Thorell et al., 2017).

We could not verify a possible influence of depressive symptoms on cognitive performance, although there was remission of symptoms in both cases.

The results are also congruent with previous findings regarding the heterogeneity of cognitive expression and intraindividual variations within an evaluation session and across sessions (Salthouse, 2007; Kuntsi and Klein, 2012; Mostert et al., 2015; Thorell et al., 2017). The cognitive profiles of the cases in the present study show differences. The first case 1 exhibited more oscillations from one assessment to another and presented impairment in only one domain (inhibitory control) at T1 and T2 but with an improvement at T3.

The second case exhibited more impaired domains, mainly in the first assessment (T1), with *z*-scores varying between -1.5 and -5.8. At T2, there was an improvement in five of the six scores altered in T1, which leads to questions about a possible subjective factor related to the impact of a lack of familiarity with the evaluation at T1. In contrast to Case 1, Case 2 presented difficulties with memory, notably, verbal late recall (RAVLT), and beyond visual immediate and late recall (BVMT). However, recognition skills in both tests presented as preserved, suggesting failure in the information search strategy rather than difficulty in storing, as has been found in studies with younger participants with ADHD (Skodzik et al., 2017). In terms of inhibitory control, there was significant improvement on the Stroop (III) test over the course of the evaluations (as was true for Case 1), which suggests practice effects as predicted in previous studies (Goldberg et al., 2015).

Although we do not know how much the patient’s cognitive performance impairments were influenced by changes due to aging in the last decade, the impairments are consistent with her lifetime complaints.

Regarding possible cognitive decline, there was no significant decline seen in the comparison between T1 and T3 in both cases. Despite fluctuations in performance, when analyzing individual results (functions that were and were not impaired), there was, in each case, a tendency toward the maintenance of each of their unique cognitive profiles over the three assessments. This tendency provides important clues to the analysis of subsequent changes in the evolution of cognitive functioning.

Due to the fluctuations, a minimum of three consecutive evaluations at baseline may be necessary to acquire a cognitive profile. We hypothesize that longer follow-up of individuals with ADHD is necessary to detect an early real cognitive decline based on objective measures. To minimize the confounding aspect of the fluctuations in clinical practice, the application of RCI may be necessary in addition to controlling for practice effects since improvement due to practice could mask subtle decline in cognition.

For comparison purposes and because a reference more suitable for considering cognitive decline in ADHD is lacking, it is instructive to look at the cutoffs proposed in the DSM-5, which range between 1.0 and 2.0 SDs from the mean for mNCD and >2.0 SDs for dementia. It is expected that some healthy elderly individuals convert to mNCD and some cases of mNCD convert to dementia, both in approximately 4 years, although most mNCD individuals do not develop dementia (Oulhaj et al., 2009; Marcos et al., 2016). Given that ADHD is a little-known condition in the elderly population and that we do not have specific normative data for this population (e.g., pathological limits, floor or ceiling effects), the clinical interpretation of extreme scores in elderly people with ADHD should be examined without precipitation and with thorough consideration of functionality over the lifetime. The development of cognitive tests that are more sensitive and ecological, including functionality scales more appropriate to this specific population, is necessary.

When we investigated the informants’ perception of possible cognitive decline using the IQCODE, the informant in Case 2 was asked to consider only the period after the start of the treatment. Despite the objective measures of the assessment, the chronic limitations in the participants’ daily functioning, and a slight decline verified through the IQCODE for Case 2, participants were active, worked, and independently performed instrumental and basic activities of daily life during the period in which they were evaluated. More compromised scores and relatively preserved functionality (as in Case 2) could be explained by other possible factors reflecting the individual’s achievement in later life to limit the impact of her decline to those few life situations in which she needs to perform at her maximum, relying more with increased age on acquired knowledge and less on solving new problems (Salthouse, 2007).

Another factor that should be considered involves the chronic use of methylphenidate, mainly in Case 2. The treatment may have positive effects on brain structure over time from child to adulthood (Frodl and Skokauskas, 2012) and may improve some cognitive functions such as working memory, interference control, processing speed, verbal learning (Biederman et al., 2012), sustained attention (Agay et al., 2014), and reaction time (Kofler et al., 2013), although one study failed to find influences on task performance (Mostert et al., 2015). However, the long-term effects of methylphenidate in older adults are not yet known. Thus, it would be interesting for future studies to investigate whether methylphenidate acts on brain structures and plasticity as a neuroprotective factor against cognitive decline, although the basis for such an investigation is speculative.

Our findings do not allow us to make consistent predictions as to whether cognitive deficits become general over time, resulting in greater global cognitive impairment, or if the difficulties in specific domains merely increase in severity over time, with greater impairment in specific situations that are part of the patients’ daily functioning. The present study does suggest that, given the complexity and heterogeneity of cognitive expression in ADHD, it will be difficult for future studies to find patterns in the level of oscillations of group performance data to establish a decline in individual cognitive abilities.

In routine clinical assessments, individual characteristics of each participant and multiple other variables unrelated to ADHD, such as the presence of vascular risk factors or pre-existing medical problems, environmental influences, and other factors that might arise over time, should also be taken into consideration. Older adults with ADHD should be subjected to periodic, broad, and thorough clinical evaluations to rule out confounding factors, allowing for appropriate differential diagnoses and enabling the establishment of early and appropriate clinical and/or environmental interventions designed on an individual basis.

## Concluding Remarks

Except for working memory performance, the cognitive profiles found in the two cases are congruent to those reported in other studies conducted in individuals with ADHD. Despite variations from one session to another, it is possible (and necessary) to identify a patient cognitive profile, which should be useful for analysis of cognitive evolution. Consistent cognitive decline was not identified in either of the participants in the 2 years of follow-up. However, these patient profiles represent possible cognitive patterns that may be found in a clinical setting, and they suggest a less pessimistic perspective about cognitive impairments that could be a prodrome of ADHD-related dementia. We also identified some aspects of neuropsychological assessments that may be useful in clinical practice as well as suggestions for individual longitudinal assessments. Further studies should employ longitudinal data, include healthy controls, and avoid the limitations of the present study.

## Ethics Statement

Participants signed a free and informed consent form for the purposes of research participation as well as for the publication of the case report, including all identifiable information/data. The final format of the manuscript along with the authorizations of publication were submitted and approved by the Ethics Committee of the Hospital das Clínicas of the University of São Paulo. Protocol Number 3.118.878.

## Author Contributions

MK conducted the neuropsychological testing and wrote the present study’s results, discussion, and conclusion. MS diagnosed and treated the patients and wrote the case descriptions. APS, GB, and ML contributed to the study design, interpretation, and review of the manuscript. All authors approved the final version of the manuscript for submission and are in accordance with regard to the accuracy and integrity of the work.

## Conflict of Interest Statement

The authors declare that the research was conducted in the absence of any commercial or financial relationships that could be construed as a potential conflict of interest.
