# Supplementary material for: Longitudinal Neuropsychological Assessment in Two Elderly Adults With Attention-Deficit/Hyperactivity Disorder: Case Report
Source: Front Psychol. 2019 May 28;10:1119. doi: 10.3389/fpsyg.2019.01119 (PMC6546833; doi:10.3389/fpsyg.2019.01119)
Supplement: Supplementary file 1 [file Data_Sheet_1.PDF]

PARECER CONSUBSTANCIADO DO CEP

DADOS DO PROJETO DE PESQUISA

Título da Pesquisa: Longitudinal neuropsychological assessment in two elderly adults with Attention Deficit Hyperactivity Disorder

Pesquisador: ANTONIO DE PADUA SERAFIM

Área Temática:

Versão: 1

CAAE: 05512818.9.0000.0068

Instituição Proponente: Hospital das Clínicas da Faculdade de Medicina da USP

Patrocinador Principal: Financiamento Próprio

DADOS DO PARECER

Número do Parecer: 3.118.878

Apresentação do Projeto:

Este é um estudo (Relato de Casos) voltado para o Deficit de Atenção e Hiperatividade no adulto

Objetivo da Pesquisa:

Visa divulgar o curso clínico e métodos subsidiários nesta entidade.

Avaliação dos Riscos e Benefícios:

Não haverá procedimentos invasivos ou outras intervenções de risco, coletando-se dados já disponíveis. Os benefícios são científicos, de aprofundamento dos conhecimentos.

Comentários e Considerações sobre a Pesquisa:

A proposta está corretamente elaborada e poderá atualizar as informações na área

Considerações sobre os Termos de apresentação obrigatória:

O Termo de Consentimento autoriza a divulgação dos achados dos pacientes.

Recomendações:

Não há

Conclusões ou Pendências e Lista de Inadequações:

Não há pendências

Considerações Finais a critério do CEP:

Endereço: Rua Ovídio Pires de Campos, 225 5º andar
Bairro: Cerqueira Cesar **CEP:** 05.403-010
UF: SP **Município:** SAO PAULO
Telefone: (11)2661-7585 **Fax:** (11)2661-7585 **E-mail:** cappesq.adm@hc.fm.usp.br

Este parecer foi elaborado baseado nos documentos abaixo relacionados:

Tipo Documento	Arquivo	Postagem	Autor	Situação
Informações Básicas do Projeto	PB_INFORMAÇÕES_BÁSICAS_DO_PROJETO_1278150.pdf	08/01/2019 10:35:25		Aceito
TCLE / Termos de Assentimento / Justificativa de Ausência	TCLERC.pdf	28/12/2018 11:59:00	ANTONIO DE PADUA SERAFIM	Aceito
Projeto Detalhado / Brochura Investigador	RC.doc	28/12/2018 11:58:37	ANTONIO DE PADUA SERAFIM	Aceito
Cronograma	Cronograma.doc	28/12/2018 11:58:07	ANTONIO DE PADUA SERAFIM	Aceito
Folha de Rosto	FR1.pdf	28/12/2018 11:56:10	ANTONIO DE PADUA SERAFIM	Aceito

Situação do Parecer:
Aprovado

Necessita Apreciação da CONEP:
Não

SAO PAULO, 23 de Janeiro de 2019

Assinado por:
ALFREDO JOSE MANSUR
(Coordenador(a))

Endereço: Rua Ovídio Pires de Campos, 225 5º andar
Bairro: Cerqueira Cesar **CEP:** 05.403-010
UF: SP **Município:** SAO PAULO
Telefone: (11)2661-7585 **Fax:** (11)2661-7585 **E-mail:** cappesq.adm@hc.fm.usp.br
